# A new family of bizarre durophagous carnivorous marsupials from Miocene deposits in the Riversleigh World Heritage Area, northwestern Queensland

**DOI:** 10.1038/srep26911

**Published:** 2016-05-27

**Authors:** M. Archer, S. J. Hand, K. H. Black, R. M. D. Beck, D. A. Arena, L. A. B. Wilson, S. Kealy, T.-t. Hung

**Affiliations:** 1 PANGEA Research Centre, School of Biological, Earth & Environmental Sciences, University of New South Wales, NSW 2052, Australia; 2 School of Environmental & Life Sciences, University of Salford, Salford M5 4WT, UK; 3 Department of Archaeology and Natural History, School of Culture, History and Language, College of Asia and the Pacific, Australian National University, ACT 2601, Australia; 4 Biological Resources Imaging Laboratory, Mark Wainwright Analytical Centre, University of New South Wales 2052, Australia

## Abstract

A new specimen of the bizarrely specialised *Malleodectes mirabilis* from middle Miocene deposits in the Riversleigh World Heritage Area provides the first and only information about the molar dentition of this strange group of extinct marsupials. Apart from striking autapomorphies such as the enormous P3, other dental features such as stylar cusp D being larger than B suggest it belongs in the Order Dasyuromorphia. Phylogenetic analysis of 62 craniodental characters places *Malleodectes* within Dasyuromorphia albeit with weak support and without indication of specific relationships to any of the three established families (Dasyuridae, Myrmecobiidae and Thylacinidae). Accordingly we have allocated *Malleodectes* to the new family, Malleodectidae. Some features suggest potential links to previously named dasyuromorphians from Riversleigh (e.g., *Ganbulanyi*) but these are too poorly known to test this possibility. Although the original interpretation of a steeply declining molar row in *Malleodectes* can be rejected, it continues to seem likely that malleodectids specialised on snails but probably also consumed a wider range of prey items including small vertebrates. Whatever their actual diet, malleodectids appear to have filled a niche in Australia’s rainforests that has not been occupied by any other mammal group anywhere in the world from the Miocene onwards.

Until recently, all known dasyuromorphians have been comfortably accommodated into one of three families: Dasyuridae, Thylacinidae and Myrmecobiidae, with the interrelationships of these clades the subject of long-standing controversy^1^,^2^,^3^,^4^,^5^,^6^. In the main, this reflects modern dasyuromorphian biodiversity because relatively little of the fossil record of the order is known, with none at all for myrmecobiids. Within dasyurids and thylacinids, dental morphology is more or less similar resulting in arguments about little more than subfamilial distinctions within existing family-level clades.

Recent discoveries being made in the Oligo-Miocene deposits of the Riversleigh World Heritage Area in NW Queensland have resulted in publication of a series of anomalous carnivorous taxa that do not fit comfortably within any of the three known dasyuromorphian families. In most cases these have been published as new genera with uncertain familial position.

Wroe^7^,^8^ and Godthelp *et al.*^9^ further argued that some fossil dasyuromorphian-like taxa (e.g., the late Oligocene *Ankotarinja* and *Keeuna*^10^) do not preserve sufficient features (e.g., complete incisor dentitions) to enable them to be placed with confidence in Dasyuromorphia, or even the cohort clade Australidelphia (which includes all living Australian marsupials plus the South American *Dromiciops*). Godthelp *et al.*^9^ classified *Ankotarinja* and *Keeuna* as Marsupialia *incertae sedis*, together with the early Eocene *Djarthia*, which has now been shown to be a stem-australidelphian^11^. Nevertheless, Wroe^12^ allocated one of the taxa referred to here, *Ganbulanyi*, based only on two isolated teeth, to Dasyuridae. The practical path in palaeontology involving fragmentary fossils is tentative allocation based on the range of morphology exhibited by crown group clades. Yet with a plethora of new fossils turning up in the Australian fossil record that possess previously unknown combinations of plesiomorphic and apomorphic features, even this approach is proving to be increasingly difficult.

The description of *Ganbulanyi djadjinguli*^12^ was based on an isolated upper tribosphenic molar of uncertain position in the tooth row and an isolated premolar similarly of uncertain position, both from the possibly latest middle or earliest late Miocene Riversleigh Faunal Zone D^13^,^14^,^15^,^16^,^17^,^18^ Encore Site Local Fauna at Riversleigh. The molar (QM F24537) was made the holotype of the species. Assignment of these two teeth to the same dasyurid taxon was based on apparent ‘bone-cracking’ (i.e., bone-crushing) adaptations and at least partial resemblance of both teeth to those of species of *Sarcophilus*.

Description of *Malleodectes mirabilis*^19^ was based on a partial left maxilla which retained P2–3 as well as partial alveoli for C1, P1, M1 and M2 from the late Middle Miocene Rick’s Rusty Rocks Site at Riversleigh^17^. The hypertrophied P3 closely resembled the premolar referred by Wroe^12^ to *G. djadjinguli*. Arena *et al.*^19^ concluded that the premolar referred by Wroe to *G. djadjinguli* probably did not represent the same species as the holotype molar (QM F24537), for three reasons. First, there was no direct evidence that these two teeth were in any way associated with each other. Second, the preserved alveolar remnants in the holotype maxilla of *M. mirabilis* could not have accommodated a molar the size of the holotype of *G. djadjinguli*, whereas the premolars of the two taxa are similar in size and shape. Third, the original interpretation of the *M. mirabilis* maxilla concluded that the tooth row exhibited a steep posterior decline in crown size from P3 to at least M3, in contrast to the hypothetical tooth row of *G. djadjinguli* where the only molar known, an incomplete tooth^12^, is much larger than P3. Similarity as well as some differences in structure of the latter to the P3 of *M. mirabilis* led Arena *et al.*^19^ to conclude it represented another species of *Malleodectes* which they named *M. moenia*. Both species of *Malleodectes* were regarded by Arena *et al.*^19^ to probably be specialised predators of gastropods and convergent in terms of their dentition on Australian snail-eating lizards (*Cyclodomorphus* spp).

A fragmentary juvenile maxilla (QM F57925) representing a species of *Malleodectes* was subsequently discovered at AL90 Site at Riversleigh (Fig. 1). AL90 speleothems have been radiometrically dated as 14.64 ± 0.47 Ma^17^ and assigned to middle to late Riversleigh Faunal Zone C^18^. In the crypt below dP3 there is an incompletely formed but distinctive P3 that closely resembles the P3 of *M. mirabilis*. However, the maxilla also retains dP3, M1, M2 and a portion of the developing alveoli for an unerupted M3. QM F57925 shows that the molar row does not steeply decline in size from front to back (see below). Reconsideration of the holotype of *M. mirabilis* indicates that most of the buccal half of the alveoli for the molars in *M. mirabilis* is missing from that specimen hence there is no convincing evidence that it differed in this respect from QM F57925, *contra* to the original interpretation. Reconsideration of this feature invites a review of the dietary hypothesis presented by Arena *et al.*^19^.

Most recently, an isolated M2 or M3 has been ascribed to a new genus and species, *Whollydooleya tomnpatrichorum* from Whollydooley Site on Whollydooley Hill, west of the Riversleigh World Heritage Area^20^. While the deposit has not been dated, factors discussed in that paper suggest it could be late Miocene in age. *Whollydooleya tomnpatrichorum* shares a few apomorphic features with the molars of species of *Sarcophilus* but other very different features (e.g., hypertrophied entoconids) led Archer *et al.*^20^ to conclude that this Miocene hypercarnivore was probably part of a separate radiation not closely related to dasyurine dasyurids. The hypercarnivorous *W. tomnpatrichorum* may be related to species of *Malleodectes*, given some of the same *Sarcophilus*-like features seen in *Ganbulanyi*, but at present there is no convincing evidence that this is the case.

The highly distinctive juvenile maxilla (Fig. 2) described in the present paper is referred to *M. mirabilis*. The morphology of the preserved C1, P3 and dP3-M2 leads us to conclude that this taxon (possibly along with *G. djadjinguli*; see below) merits establishment of a new, highly distinctive family. Qualitative comparisons and formal phylogenetic analysis suggest that this family is probably a member of Dasyuromorphia (although this is not certain) and it does not appear to share an exclusive relationship with Dasyuridae, Myrmecobiidae or Thylacinidae.

Postcanine cheektooth homology used here follows previous work^20^,^21^ such that dasyuromorphian marsupials have one deciduous tooth, dP3, and four molars. Tooth cusp and blade nomenclature follows Archer^22^,^23^ and Archer *et al.*^20^. Higher level systematic nomenclature follows Aplin and Archer^24^, Beck^25^ and Beck *et al.*^26^.

## Results

Systematic Palaeontology

Subclass: Marsupialia Illiger, 1811

Order: Dasyuromorphia Gill, 1872

Family Malleodectidae nov.

**Included genera:** *Malleodectes* Arena *et al.*^19^

### Familial diagnosis

Medium-sized (~1 kg; see below), durophagous, carnivorously-adapted marsupials that differ from all others in the following combination of features: large, caniniform, laterally compressed C1; narrow, premolariform P1 adpressed against the base of C1; asymmetric P2 with wide, diamond-shaped (in occlusal view) posterior region, posteriorly-sloping crown and low, narrow, attenuated anterior region; uniquely (among known dasyuromorphians) large dP3 (similar in size to M1) with three cusps and a functional postmetacrista; enormous, subrounded, dome-shaped, essentially unicuspid, four-rooted P3 that is wider and longer than M1 and M2 (and probably M3), with (*M. moenia*) or without (*M. mirabilis*) a tiny cuspule near the posterior edge of the crown; M1 relatively (compared to M2) hypsodont, longer and wider than M2, with StB and StD directly buccal to the paracone and metacone respectively, StD taller than StB, a deep vertical fissure on the buccal flank of the crown, no anterior ectoloph crest, StE present on posterior ectoloph ridge, poorly-developed straight (M1) centrocrista and no posterior cingulum; M2 more conventionally dasyuromorphian-like with v-shaped centrocrista but with conules better developed than in most undoubted dasyuromorphians.

### Etymology

The family name derives from the type genus *Malleodectes*^19^.

### Materials

In addition to specimens noted by Arena *et al.*^19^ we describe here QM F57925, juvenile cranial material including fragmentary left nasal and? frontal bones and a left maxilla with C1, P1, dP3, P3 crown (unerupted), M1–2, alveoli for P2 and M3. QM F57925 is from AL90 Site, a middle Miocene deposit that has been radiometrically dated as 14.64 ± 0.47 Ma old, and which contains a fauna correlating with mid- to late- Riversleigh Faunal Zone C^17^,^18^,^19^,^27^. AL90 has been interpreted to be a cave deposit, the original entrance of which acted as a natural pit-fall trap^27^,^28^,^29^.

### Estimated body mass

Using the “dasyuromorphian-only” dataset of Myers^30^, the most accurate regression equation that can be used to calculate body mass for *Malleodectes mirabilis* is the occlusal area of M2. This gives an estimated body mass (including the smearing estimate) of 896 g.

### Description of QM F57925, *Malleodectes mirabilis*

**Figure 3:**
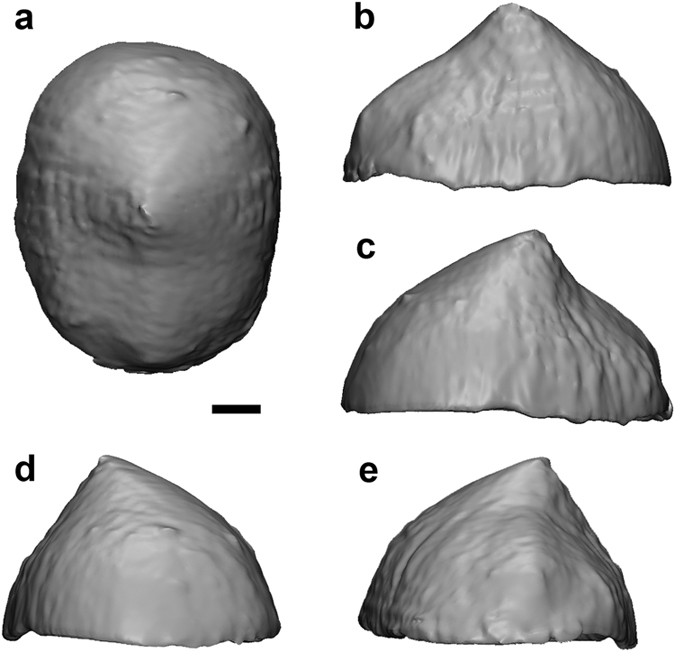
The unerupted left P^3^ of QM F57925. Images are captured from the 3D model, which was manually segmented from DICOM images (micro Computed Tomography [micro-CT] scan), and show the specimen in (**a**) occlusal view, (**b**) buccal view, (**c**) lingual view, (**d**) posterior view and (**e**) anterior view. The length of this P^3^ is 6.7 mm; its maximum transverse width is 5.4 mm. Scale bar = 1 mm.

**Figure 4:**
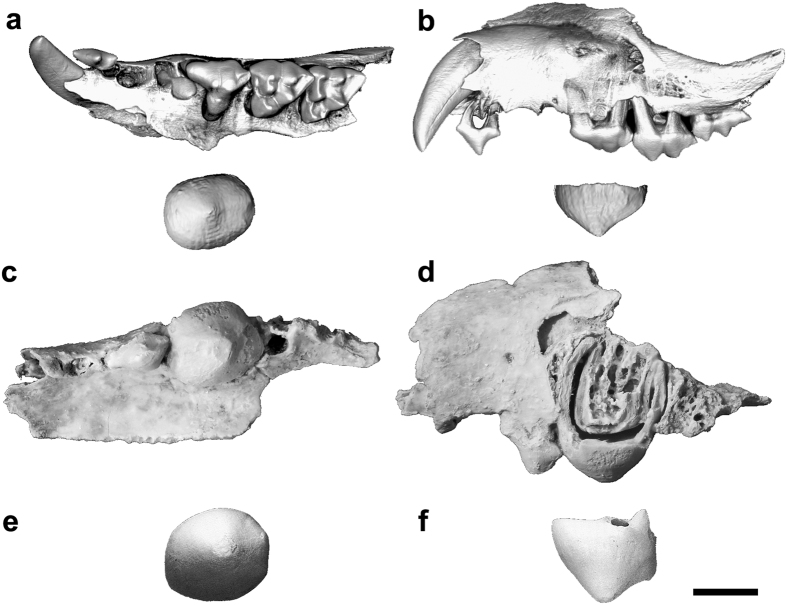
Specimens of *Malleodectes* species. All specimens are to scale and aligned at the P3/M1 boundary. Images show (**a**) *M. mirabilis* left maxilla and unerupted left P3 crown ‘extracted’ from microtomographic scan, occlusal view (QM F57925), (**b**) *M. mirabilis* left maxilla and unerupted left P3 ‘extracted’ from micro-CT scan, buccal view (QM F57925), (**c**) *M. mirabilis* left maxilla, occlusal view (QM F50847, holotype), (**d**) *M. mirabilis* left maxilla, buccal view (QM F50847, holotype), (**e**) Mirror reversed image of *M. moenia* right P3, occlusal view (QM F30464) and (**f**) Mirror reversed image of *M. moenia* right P3, buccal view (QM F30464). Scale bar = 5 mm.

QM F57925 is identified here as *Malleodectes mirabilis* and is differentiated from *M. moenia* based on the following features of P3: absolutely smaller; less rounded; lower-crowned; and lacking a posterior cuspule^19^. Comparison of P3 with that of the holotype of *M. mirabilis* (QM F50847) and that which Wroe^12^ originally referred to *Ganbulanyi djadjinguli* but which Arena *et al.*^19^ identified as *M. moenia*, was enabled through digital extraction of the unerupted P3 from micro-CT images (Fig. 3). All P3s referred here to species of *Malleodectes* are compared in Fig. 4. P3 of QM F57925 is 5.4 mm wide, 6.7 mm long and most closely approximates P3 in *M. mirabilis* (5.6 wide; 6.5 mm long) rather than the larger *M. moenia* (6.5 mm wide; 7.1 mm long).

To the description provided by Arena *et al.*^19^ of the P2 and P3 of the holotype of *M. mirabilis,* we add the following.

Cranial fragments. Left nasal isolated but relatively complete. Bounded medially by suture facing right nasal, posteriorly by naso-frontal suture. Lateral edge broken and missing sutural contact with left maxilla. Second cranial fragment may be portion of left frontal but sutural relationships unclear.

Maxilla. Lateral wall of preserved section retains maxilla-jugal suture. Position of ventral rim of orbit not clear. Anterior rim of orbit did not extend forward beyond level of anterior margin of M1. Infraorbital canal intact and located above anterior root of M1. It emerges onto lateral wall of rostrum via two separate foramina (larger dorsal, and much smaller ventral foramina) housed entirely in maxilla. Distinct trough evident for maxillary branch of trigeminal nerve and/or associated artery. Trough ascends maxillary wall as it extends forward, presumably to circumvent tumescent lateral maxillary bulge caused by unerupted huge P3 crown. Preserved palatal section of maxilla missing anterior medial margin. Lacrimal contribution to dorsal rim of maxillary foramen uncertain based on preserved orbital portion of maxilla. Palatal edge of maxilla medial to M1 represents part of lateral margin of a maxillary palatal vacuity^31^ of unknown size.

C1. Large, caniniform, partially erupted (no higher occlusally than P1), but likely clearly prominent, when fully-erupted. Absence of posterior blade suggests primary function to puncture rather than cut. Proximal end of C1 root narrow, excluding possibility of hypselodonty. Position of canine alveolus, relative to maxilla- premaxilla suture, unclear (occurs in association with maxillary-premaxilla suture in holotype^19^ rather than solely within maxilla such as occurs in dasyurids, myrmecobiids, and several peramelemorphians).

No diastema between any cheekteeth possibly reflecting developmentally juvenile individual, hence more brachycephalic and brevirostral individual than holotype^32^.

P1. Unicuspid, simple, lacking basal cingula, additional cuspules or blades. Anterior root slender, curves posterodorsally. Posterior root larger, rounder, less (if at all) curved. P1 primary function (as in C1) to puncture and hold.

P2. Here only alveoli preserved but P2 is present in holotype. Alveoli suggest anterior root round and larger than posterior root which was transversely wider than long. Orientation of alveoli indicates anterior and posterior roots converged distally. Posterior wall of posterior alveolus exhibits fissure seemingly related to adjacent unerupted P3. P2 and dP3 may be displaced or forced out of alignment in some individuals by eruption of enormous P3 whose unerupted crown partly overlaps posterior root of P2, although P2 remains intact in the holotype^19^. Variation in retention of P2 is a feature of the propleopine hypsiprymnodontid *Ekaltadeta ima* which also has a massively hypertrophied P3^33^. At least temporary retention of P2 after P3 erupts and displaces dP3 is unique among macropodiforms to hypsiprymnodontids^34^.

dP3. Tricuspid with large metacone, smaller paracone and much reduced protocone. dP3 extremely large for a dentally relatively plesiomorphic marsupial. In terms of meristic gradients, absolute size and several morphological features, dP3 mimics M1 except in lacking a well-developed stylar shelf. Of three primary cusps, metacone is slightly larger than slightly smaller, closely adpressed and basally joined paracone, both of which are much larger than the protocone. Pronounced vertical fissure separates buccal flanks of paracone and metacone. No stylar cusps present. Paracone anterior flank descends towards anterobuccal corner of crown without interruption. Paracone lacks subtending blades. Metacone mimics that cusp in M1, with large, longitudinally orientated, functional postmetacrista that descends posteriorly from apex to posterior extremity of crown. This blade parallels M1 postmetacrista and presumably formed a scissorial blade pair with paracristid of m1. Similarly functional postmetacrista is observed on dP3 of some dasyurids (e.g., *Sminthopsis* spp.), but in many others and also *Thylacinus cynocephalus* dP3 is vestigial without distinct cusps or crests. Rudimentary, low stylar shelf developed on posterobuccal flank of crown. Its posterior margin mimics structure in M1 in having a distinct but low posterobuccal rim that loops around the posterior corner of the crown to become confluent with postmetacrista. Protocone very reduced, closely adpressed to base of metacone. Incomplete, very reduced preprotocrista extends for short distance across anterior base of metacone.

P3. Unerupted, incompletely developed (lacking roots), relatively enormous tooth with dome-like crown topped by small, pointed peak. Posterior cuspule not evident. Root number indeterminate as they have yet to form. P3 of *M. moenia* displays four roots^19^. Root number in holotype P3 of *M. mirabilis* also indeterminate, but likely four given presence of a bulge near the posterolingual crown base suggestive of presence of smaller lateral roots (as in *M. moenia*) in addition to large anterior and posterior roots. Given the interpreted durophagous use of hypertrophied P3, the extra small buccal and lingual roots presumably helped stabilize the crown when hard objects were being crushed.

M1. Slightly displaced (post-mortem) above alveolar plane. More conventionally tribosphenic than dP3 when compared with M1 of dasyurids but overall robust and semi-bunodont (compared with M2). Structure suggests capacity to sustain considerable occlusal pressure when biting down on hard materials. There are six cusps (in order of decreasing height): metacone, StD, paracone=StB, StE, protocone. Flanks and bases of StB and StD expand buccally towards crown base resulting in proportionately taller and wider buccal flank in contrast to shape of M2. Bilobate in occlusal view. Well-developed vertical groove separates flanks of StB and StD from top to base. Subequal paracone and StB united by tall, narrow preparacrista. Poorly-developed postparacrista ridge descends posterobuccal flank of paracone. Premetacrista vestigial. Consequently, a fully functional centrocrista uniting the paracone and metacone is absent, and hence M1 is not conventionally dilambdodont. However, a low, poorly-defined ‘bridge’ unites the posterior end of the postparacrista and anterior flank of the metacone. Similarly, a functional buccal ectoloph is absent yet poorly-developed ridges occupy the posterior and anterior flanks of StB and StD, respectively. Postmetacrista development more conventional relative to dasyuromorphians, extending to posterior corner of crown where it meets low posterior ridge from StD. These blades define a posterior buccal basin. No blades or buttresses unite the metacone and StD. Tiny StE apparent on ectoloph midway between StD and metastylar corner of crown. Protocone narrow, low, but massive with wide base. Preprotocrista distinct but small. Extends across anterior flank of paracone as a distinct anterior cingulum. Terminates below StB, before reaching parastylar corner. Inflection in preprotocrista may represent poorly defined protoconule. Postprotocrista and posterior cingulum absent. Protocone separated from base of metacone by distinct cleft. Lingual protocone root abnormally diminished failing to occupy entire alveolus. May indicate a pathological gum/maxillary condition given that the lingual root of M2 is normal. Alternatively, it may be the result of eruptive processes involving P3 whose posterior margin in the unerupted state underlies the anterior region of M1.

M2. Incompletely erupted, tribosphenic (more so than M1) tooth with nine cusps (in order of decreasing height): metacone > StD > paracone = StB > StA = StE > protocone > protoconule = metaconule. Buccal flank of crown much lower than that of M1 hence more conventional in form. High, distinct preparacrista unites paracone and StB. Poorly-developed but distinct ectoloph ridge unites StB and StD. Medial inflection in ectoloph occurs above a vertical groove that ascends the buccal crown flank, dividing it into shorter anterior and longer posterior sections (this groove is more pronounced in M1 dividing crown into equally-long anterior and posterior sections). Transverse ridge-like swelling between metacone and StD; divides the area between stylar cusps and paracone and metacone into a rectangular, thinly-enameled anterior basin and a triangular posterior basin. These are buccal to primary blades hence their primary function may be to prevent food items from slipping off the occlusal platform. Small StE evident on posterior section of ectoloph ridge (closer to StD than posterior corner of crown). Moderately v-shaped centrocrista formed by short distinct postparacrista and long premetacrista. Distinct metaconule and smaller distinct protoconule occupy postprotocrista and preprotocrista, respectively, and with protocone, completely enclose a protoconal basin. Well-formed anterior cingulum extends from buccal end of preprotocrista to base of StA. M1 posterior extremity inserts into anterior notch between parastylar corner and buccal end of M2 anterior cingulum. This notch minimizes transverse independent movement of M1 and M2. Posterior cingulum absent although a row of small swellings evident along posterobuccal base of crown below lingual half of the postmetacrista.

M3. Not preserved, but remnant alveoli indicate it was just beginning to erupt and almost certainly not yet in occlusion with lower dentition. Alveolar area indicates M3 large, as long (possibly longer), and at least as wide as M2. Posterior flank would have been distinctly longer than anterior flank.

Measurements are given in Table 1.

### Phylogenetic analysis

**Figure 5:**
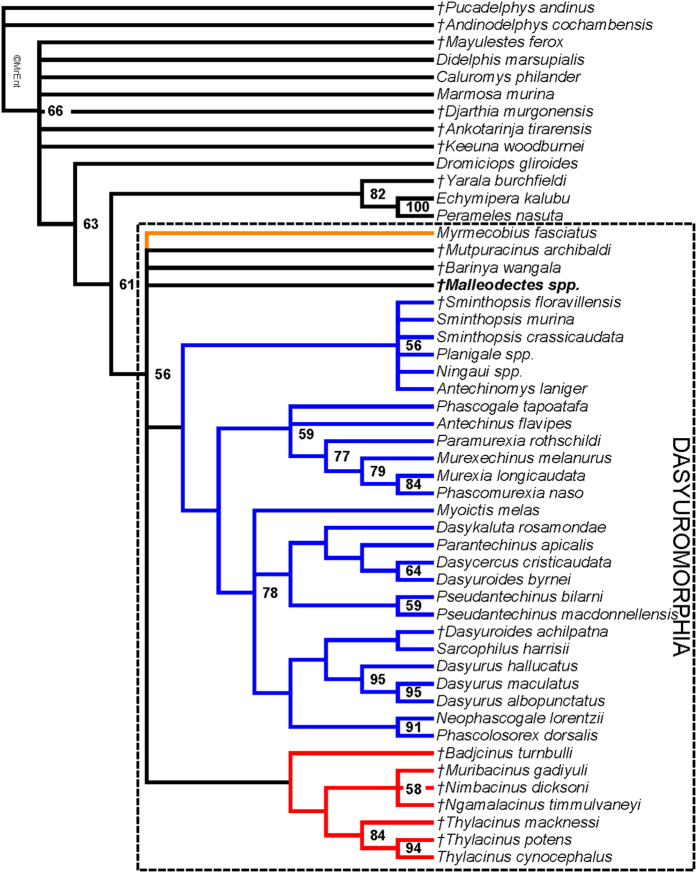
Strict consensus of 18619 most parsimonious trees (length = 340 steps; CI excluding autapomorphies = 0.393; RI excluding autapomorphies = 0.616) resulting from maximum parsimony analysis of the 62 craniodental character matrix, with relationships among modern taxa constrained to match recent molecular studies^5^,^6^,^46^. Values at nodes represent bootstrap values > 50%. Fossil taxa are indicated by daggers (†), with *Malleodectes* highlighted in bold. The three currently recognised dasyuromorphian families are coloured: Dasyuridae in blue, Myrmecobiidae (of which *Myrmecobius fasciatus* is the sole known representative) in orange and Thylacinidae in red.

Maximum parsimony analysis of the 62 craniodental character matrix, with relationships among modern taxa constrained to match recent molecular studies, found 18619 most parsimonious trees (length = 340 steps; CI excluding autapomorphies = 0.393; RI excluding autapomorphies = 0.616). A strict consensus of these is shown in Fig. 5. *Malleodectes* is recovered within Dasyuromorphia, albeit with weak support (bootstrap = 56%). Within Dasyuromorphia, *Malleodectes* forms a basal polytomy with the only known myrmecobiid *Myrmecobius*, the fossil *Mutpuracinus* (identified in previous studies as a thylacinid^35^,^36^), the fossil *Barinya* (described as the most plesiomorphic known dasyurid^37^) and two weakly-supported clades corresponding to Dasyuridae and Thylacinidae.

## Discussion

The species of *Malleodectes* are among the most extraordinarily distinctive Australian carnivores known. We consider that it is probably a dasyuromorphian rather than a representative of some other order of marsupicarnivores for the following reasons. First, StD is larger than StB on both M1 and M2, which is the common condition in dasyuromorphians, but is rare amongst other marsupials. Second, there is no ‘central cusp’ lingual to the ectoloph on M1 or M2 such as occurs in the enigmatic late Oligocene marsupials *Ankotarinja* and *Keeuna* from the late Oligocene Ditjimanka Local Fauna of central Australia^10^ and the stem-australidelphian *Djarthia* from the early Eocene Tingamarra Local Fauna^9^. Third, except for its huge size, dP3 of *Malleodectes* broadly resembles those of some dasyurids (such as *Sminthopsis* spp.) in that the protocone is very small and closely adpressed to the base of the metacone, a distinct, functional postmetacrista is present, but a well-developed stylar shelf and stylar cusps are absent; in some other dasyuromorphians (many dasyurids, *Thylacinus cynocephalus*), however, dP3 is vestigial and featureless. In most modern didelphids and in most fossil tribosphenic marsupialiforms for which the deciduous dentition is known, dP3 more closely resembles the permanent molars in size and in having a well-developed protocone, distinct centrocrista, well-developed paracone and metacone and well-developed stylar shelf with distinct stylar cusps^38^,^39^.

Congruent with our qualitative assessment, the phylogenetic analysis supports dasyuromorphian affinities for *Malleodectes*. However, support for Dasyuromorphia including *Malleodectes*, and for most other clades within the phylogeny, is weak. This is likely due to two major factors. First, several of the taxa included in our analysis are known from very incomplete dental remains (often, only molars); second, *Myrmecobius fasciatus* (the only known representative of Myrmecobiidae) is characterised by a degenerate dentition, particularly the molars, and hence cannot be meaningfully scored for many dental characters. As a result, relatively few characters can be scored across all taxa, and thus there is a dearth of potential synapomorphies. Discovery of more complete remains of *Malleodectes* and other fossil dasyuromorphians (particularly plesiomorphic myrmecobiids that retain a functional dentition) will likely improve support values and clarify relationships within Dasyuromorphia. Voss and Jansa^40^ noted that loss of the posterior cingulid on the lower molars optimises as a synapomorphy of crown-clade Marsupialia, but dasyuromorphians (with the possible exception of *Myrmecobius*, which cannot be meaningfully scored for this character) have secondarily reacquired a posterior cingulid. If lower molars of *Malleodectes* are discovered and found to have a posterior cingulid, this would increase the likelihood that it is indeed a dasyuromorphian. However, the stem-australidelphian *Djarthia* and its possible relatives *Ankotarinja* and *Keeuna* also have posterior cingulids on their lower molars, suggesting that this structure has been redeveloped at least twice in marsupial evolution.

The combination of unusual dental specialisations found in *Malleodectes* – namely its hypertrophied P3, the enormous dP3, narrow canine, unusual morphology of P2 and robust nature of M1 – is not found in representatives of any of the three dasyuromorphian families described to-date (Dasyuridae, Myrmecobiidae and Thylacinidae), and it lacks obvious synapomorphies that would specifically link it to one of these three families. Our phylogenetic analysis likewise fails to unambiguously group it with a specific clade within Dasyuromorphia, instead placing it in a basal polytomy within the order. For these reasons we do not hesitate to erect a new family, Malleodectidae, to accommodate this genus. There are, however, three other fossil dasyuromorphians from Riversleigh that may possibly be more closely related to *Malleodectes* than to other members of Dasyuromorphia.

The first of these is *Ganbulanyi djadjinguli* Wroe, 1998^12^, from the possibly early Late Miocene Encore Site, a taxon based on a fractured, worn, isolated upper molar of uncertain position in the toothrow (QM F24537, the holotype) and an isolated premolar (QM F20464, a referred specimen) which Arena *et al.*^19^ concluded was P3. While the P3 is an undoubted malleodectid and was renamed *Malleodectes moenia*^19^, the relationships of *G. djadjinguli*, considering the damaged holotype molar only, are not clear. Wroe^12^, noting the strongly buttressed molar base, apical wear on the principal cusps, approximation of StB to the paracone and StD to the metacone, and longitudinally orientated postmetacrista that at least paralleled features seen in species of *Sarcophilus*, suggested it was a ‘bone-cracking’ (i.e., bone-crushing) dasyurid convergent on, but nevertheless probably not closely related to, species of *Sarcophilus*. Wroe^12^ also noted structural similarities between the referred premolar and P2 in *S. harrisii*. However, differences distinguishing *G. djadjinguli* from species of *Sarcophilus* include the wider stylar shelf that retains StC and StE in addition to StB and StD. The stylar shelf in *M. mirabilis* is wide and retains the same number of stylar cusps present in *G. djadjinguli* (albeit a plesiomorphic feature), has a similarly reduced centrocrista and both taxa appear to have been specialised for mastication of hard prey, and so a relationship to malleodectids is possible. However, features distinguishing the upper molar of *G. djadjinguli* from those of *M. mirabilis* do not support a close relationship. These include in *G. djadjinguli* the much narrower stylar shelf, larger StC, lack of StE, greater approximation of the paracone to the metacone, more closely adpressed StB to the paracone and StD to the metacone, lack of a protoconule, lack of an anterior cingulum, and, perhaps most striking, an elongate, longitudinally-orientated postmetacrista. Future discoveries of better specimens referable to *G. djadjinguli* will be required before any potential relationships to malleodectids can be more confidently assessed.

Second, Archer *et al.*^20^ described, on the basis of a damaged isolated lower molar, a new genus of dasyuromorphian as a *Sarcophilus*-size hypercarnivore (i.e., it was larger than *G. djadjinguli* and much larger than malleodectids) from the possibly late Miocene Wholly Dooley Site west of the Riversleigh World Heritage Area^20^. Its relationships to other dasyuromorphians, including other durophagous carnivores such as malleodectids, are unclear. Features that suggest it had a powerful bite include the short, wide, strongly-buttressed crown, hypertrophied protoconid, reduced metaconid and anteroposteriorly foreshortened talonid. Because lower molars for undoubted malleodectids have not been described, it is not yet possible to directly compare the features of the holotype of this new dasyuromorphian to malleodectids. Discovery of more complete lower and upper molars for *W. tomnpatrichorum* will be required to resolve the relationships of this taxon.

Third, *Barinya wangala* was named by Wroe^37^ on the basis of many specimens collected from a range of Miocene sites at Riversleigh. It somewhat resembles species of *Malleodectes*, at least superficially, in the presence of a hypertrophied P3. Although this tooth is not remotely as enlarged or multirooted in *B. wangala* as it is in species of *Malleodectes*, it is much wider than that tooth in most dasyurids and also lacks the posterior blade extending down the tooth crown that is characteristic of most dasyuromorphians (with the exception of species of *Malleodectes*) as well as other tribosphenic marsupials such as didelphimorphians and peramelemorphians. Whether this indicates possible links between barinyaines and malleodectids is unclear because these two probably correlated features of the P3 could readily be convergent in the two groups. *Barinya wangala* was originally described as the most plesiomorphic known dasyurid^37^, and the only known representative of the extinct dasyurid subfamily Barinyainae. However, our phylogenetic analysis failed to group it with crown-clade dasyurids, instead placing it in a basal polytomy within Dasyuromorphia that also included *Malleodectes*. Discovery of more complete material for malleodectids will be required before any potential close relationship with *Barinya* can be resolved.

Arena *et al.*^19^, on the basis of a less-well preserved maxilla (QM F50847) that retained P2–3 and some of the lingual alveoli for some of the molars, concluded that the molars of *Malleodectes mirabilis* may have steeply declined in size posteriorly. On the basis of the new specimen described here, QM F57925, which retains M1–2 and part of the alveoli for M3, it would appear that the molars are not steeply decreasing in size although the dimensions of M2 are slightly smaller than those of M1. From alveolar sizes, it seems probable that M3 was larger than M2. The slightly larger size of M1 than M2 may well relate to hypertrophy that has occurred in the region of dP3/P3. Clearly the molars were an important part of the overall function of the malleodectid dentition whatever foods these strange marsupials ate.

**Figure 6:**
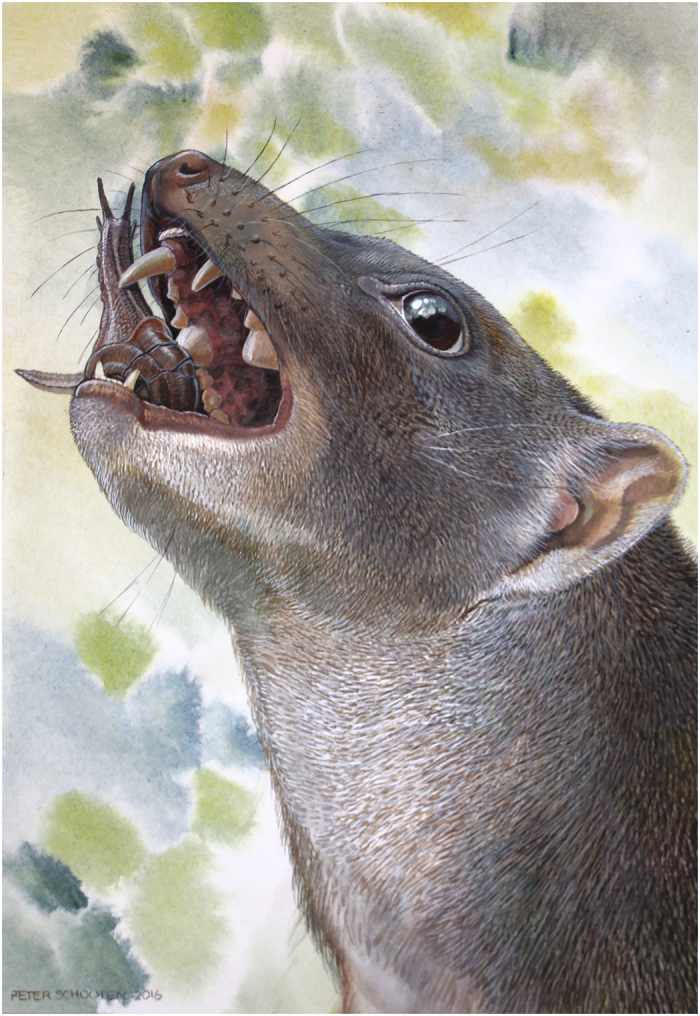
*Malleodectes mirabilis* using its massive, ball-peen-like P3 to break into what were perhaps one of this unique Miocene marsupial’s favourite meals—Riversleigh escargots. Illustration by Peter Schouten.

Based on comparison with small, heterodont, omnivorous Australian scincid lizards in the genus *Cyclodomorphus* that have a huge premolar-like tooth followed by much smaller teeth, Arena *et al.*^19^ hypothesised that, like these lizards, species of *Malleodectes* had dental adaptations for crushing the hard shells of snails. Given the thick enamel and pestle- or ball-peen hammer-like morphology of the enormous P3, the sturdy bracing by multiple long, stout roots beneath all sides of the dome-like crown of P3, the apical wear on the primary cusps of M1 as well as P3, the original interpretation of this functionality has not changed (Fig. 6). However, now that we realise the molars were more substantial than originally hypothesised, and complete with conventional, vertically-shearing blades, it is seems probable that they also consumed a wider range of prey. Although it is possible they were ossivorous (eating entire vertebrates with bony skeletons) as well as snails, the suite of dental adaptations commonly present in ossivorous mammals such as hyaenids and *Sarcophilus* is quite different. None of the latter has massively hypertrophied, dome-like crushing posterior premolars, as well as narrow canines and narrow, delicate, seemingly fragile first premolars.

Among other metatherians with enlarged premolars, North American Cretaceous stagodontids (e.g., species of *Eodelphis*, *Didelphodon*) show some at least superficial similarities to malleodectids in that they have crushing premolars followed by tribosphenic molars^41^. However, their premolars are not rounded, dome-like structures that lack basal cingula and in each of the upper and lower dentition quadrants there are two crushing premolars rather than one. Further, stagodontids have powerful canines^42^ quite unlike the narrow, far more delicate canines of malleodectids. Szalay^43^ speculated that stagodontids may have been semi-aquatic which at least raises an interesting possibility in relation to the locomotory habits of malleodectids, particularly given the interpretation that they were probably using their premolars to crush snails which are common in aquatic environments. However, Fox and Naylor^41^ contest putative evidence for the possibility that stagodontids were aquatic. In the case of malleodectids, although anything is possible, given that all malleodectid fossils found to date have come from fossil cave deposits and none from any of the aquatic deposits in Riversleigh, an aquatic lifestyle would seem less likely than a terrestrial one.

On balance, malleodectids appear to have occupied, for mammals, a unique feeding and environmental niche of their own, one not occupied by any other living or fossil mammal known.

## Conclusion

Although malleodectids are only known on the basis of partial upper dentitions, they clearly represent one of the most distinctive groups of marsupials yet discovered. Because they are known from such limited material and because of the many autapomorphic features they exhibit, relatively few of the preserved features clarify their phylogenetic relationships. The features that are available (most obviously, the larger size of stylar cusp D relative to stylar cusp B on M1–2), suggest, albeit tentatively, that malleodectids are dasyuromorphians. Our phylogenetic analysis confirms this assessment, placing *Malleodectes* within Dasyuromorphia in a polytomy that also includes dasyurids, thylacinids, the fossil forms *Barinya* and *Mutpuracinus*, and the sole known myrmecobiid *Myrmecobius fasciatus*. In terms of dental function, the well-developed molar dentition of *Malleodectes* suggests that it ate more diverse foods than just snails. The blades on the molars as well as the wear on anterior teeth suggest that small vertebrates were also part of the malleodectid diet. In combination, the large but laterally compressed C1, laterally compressed, delicate P1, hypertrophied, hammer-like P3 and tribosphenic molars with oblique shearing blades suggest that they occupied a niche in Australia’s Miocene rainforests that no other known mammalian group has managed to occupy since.

## Methods

### Numbering and measurement

Specimen numbers assigned and referred to in the text belong to the Queensland Museum (QM). All measurements provided in this study (excluding those of the unerupted P3, see below) were captured using a Leica Wild 5MA stereomicroscope with Wild MMS235 digital length measuring set.

### Computed Tomography and segmentation

Specimen QM F57925 was visualized with the aid of Micro Computed Tomography (micro-CT). The specimen was micro-CT scanned using a Siemens Inveon MicroPET-CT scanner, housed at the University of New South Wales. Slices were extracted at a thickness of 0.028 mm (total = 1467 slices) and exported as DICOM files. DICOM files were imported into Mimics ver. 18.0 (Materialise) for visualization and processing. The unerupted P3 was manually segmented from the maxilla and converted into a 3D mask. The 3D mask was exported to 3-Matic ver. 10.0 (Materialise) and linear measurements were extracted from the mask using the measurement tool.

### Phylogenetic analyses

A quantitative analysis of the phylogenetic relationships of *Malleodectes* was carried out using a matrix of discrete craniodental characters, modified from Wroe *et al.*^44^, Wroe and Musser^45^ and Murray and Megirian^36^. All 77 characters from these original studies were reassessed and rescored, with those that could not be scored confidently either redefined or deleted, and three new characters were added, resulting in a total of 62 characters in the revised matrix. Seventeen characters representing plausible morphoclines were ordered. Taxon sampling was also expanded from Wroe *et al.*^44^, Wroe and Musser^45^ and Murray and Megirian^36^, resulting in a total of 50 taxa (including *Malleodectes*), with the Paleocene stem-marsupials *Pucadelphys* and *Andinodelphys* specified as outgroup taxa. The character descriptions, matrix in NEXUS format and molecular scaffold are available as supplementary data.

The matrix was analysed using maximum parsimony, as implemented in PAUP* 4.0b10, enforcing a “backbone molecular scaffold” among living taxa based on recent molecular studies^5^,^6^,^46^. The tree search was heuristic, comprising 1000 random addition replicates. Bootstrap values were calculated using 250 replicates, saving a maximum of 1000 trees per replicate.

## Additional Information

**How to cite this article**: Archer, M. *et al.* A new family of bizarre durophagous carnivorous marsupials from Miocene deposits in the Riversleigh World Heritage Area, northwestern Queensland. *Sci. Rep.* **6**, 26911; doi: 10.1038/srep26911 (2016).

## Supplementary Material

Supplementary Information

Supplementary Dataset 1

## Figures and Tables

**Figure 1:**
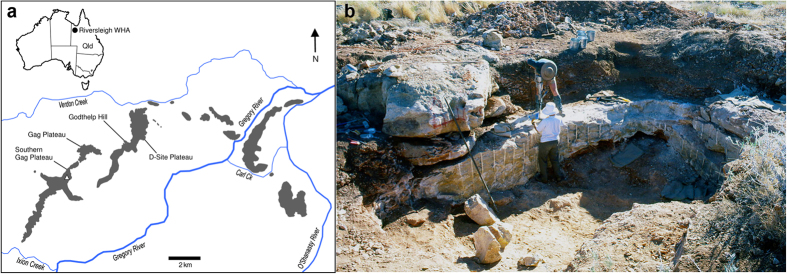
Source of *Malleodectes mirabilis*. (**a**) The Riversleigh World Heritage Area in northwestern Queensland. The white triangle indicates the position of AL90 Site. Map source: authors’ field survey data and National ASTER Map of Australia^47^. (**b**) Excavation of AL90 Site, the middle Miocene cave deposit that produced QM F57925, the left maxilla of *M. mirabilis*.

**Figure 2:**
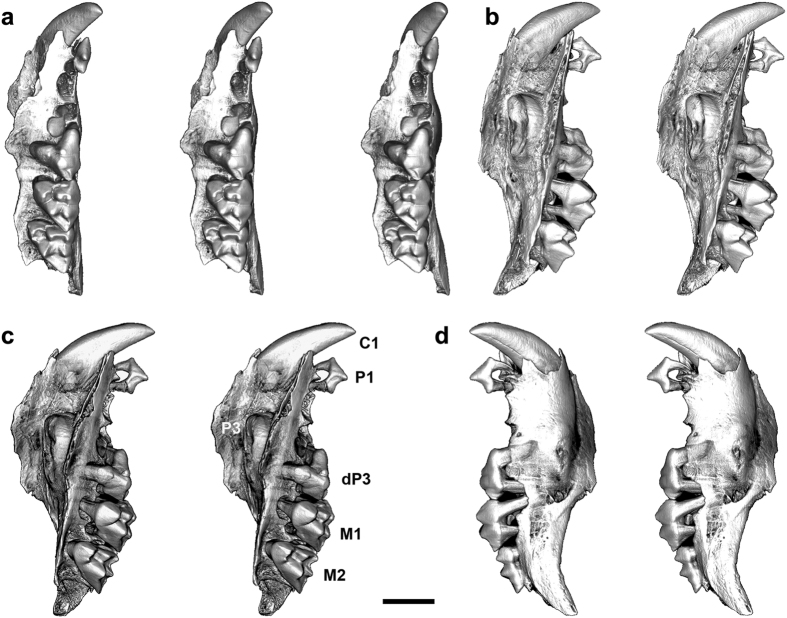
*Malleodectes mirabilis* left maxilla (QM F57925). Specimen shown in (**a**) occlusal view, stereo triplet; (**b**) lingual view, stereo pair; (**c**) oblique lingual view, stereo pair; and (**d**) buccal view, stereo pair. Scale bar = 5 mm.

**Table 1:** Measurements (mm) of QM F57925, left maxillary fragment of *Malleodectes mirabilis* from AL90 Site.

Tooth	dP3	P3	M1	M2	M3*
Max length	4.51	6.65	5.03	4.63	~4.68
Max width	4.30	5.38	4.61	4.62	>4.55

^*^Measurements for M3 are based on estimates of minimal size determined on the basis of the alveoli for this tooth.

Length C1 to post. edge of M3 alveolus: 26.23 mm; length P1 to M2, 20.54 mm.
