# Corrigendum: A new family of bizarre durophagous carnivorous marsupials from Miocene deposits in the Riversleigh World Heritage Area, northwestern Queensland

**DOI:** 10.1038/srep32054

**Published:** 2016-09-07

**Authors:** M. Archer, S. J. Hand, K. H. Black, R. M. D. Beck, D. A. Arena, L. A. B. Wilson, S. Kealy, T.-t. Hung

Scientific Reports 6: Article number: 2691110.1038/srep26911; published online: 05 27 2016; updated: 09 07 2016

The original version of this Article contained the genus and species name of an unpublished taxon ‘Whollydooleya tomnpatrichorum’. In the Introduction section:

“Most recently, an isolated M2 or M3 has been ascribed to a new genus and species, *Whollydooleya tomnpatrichorum* from Whollydooley Site on Whollydooley Hill, west of the Riversleigh World Heritage Area^20^. While the deposit has not been dated, factors discussed in that paper suggest it could be late Miocene in age. *Whollydooleya tomnpatrichorum* shares a few apomorphic features with the molars of species of *Sarcophilus* but other very different features (e.g., hypertrophied entoconids) led Archer *et al*.^20^ to conclude that this Miocene hypercarnivore was probably part of a separate radiation not closely related to dasyurine dasyurids. The hypercarnivorous *W. tomnpatrichorum* may be related to species of *Malleodectes*, given some of the same *Sarcophilus*-like features seen in *Ganbulanyi*, but at present there is no convincing evidence that this is the case”.

Now reads:

“Most recently, an isolated M2 or M3 has been ascribed to a new genus and species, from Whollydooley Site on Whollydooley Hill, west of the Riversleigh World Heritage Area^20^. While the deposit has not been dated, factors discussed in that paper suggest it could be late Miocene in age. This taxon shares a few apomorphic features with the molars of species of *Sarcophilus* but other very different features (e.g., hypertrophied entoconids) led Archer *et al*.^20^ to conclude that this Miocene hypercarnivore was probably part of a separate radiation not closely related to dasyurine dasyurids. This hypercarnivorous taxon may be related to species of *Malleodectes*, given some of the same *Sarcophilus*-like features seen in *Ganbulanyi*, but at present there is no convincing evidence that this is the case.”

In the Discussion section:

“Second, Archer *et al*.^20^ described, on the basis of a damaged isolated lower molar, a new genus of dasyuromorphian as a *Sarcophilus*-size hypercarnivore (i.e., it was larger than *G. djadjinguli* and much larger than malleodectids) from the possibly late Miocene Wholly Dooley Site west of the Riversleigh World Heritage Area^20^. Its relationships to other dasyuromorphians, including other durophagous carnivores such as malleodectids, are unclear. Features that suggest it had a powerful bite include the short, wide, strongly-buttressed crown, hypertrophied protoconid, reduced metaconid and anteroposteriorly foreshortened talonid. Because lower molars for undoubted malleodectids have not been described, it is not yet possible to directly compare the features of the holotype of this new dasyuromorphian to malleodectids. Discovery of more complete lower and upper molars for *W. tomnpatrichorum* will be required to resolve the relationships of this taxon”.

Now reads:

“Second, Archer *et al*.^20^ described, on the basis of a damaged isolated lower molar, a new genus of dasyuromorphian as a *Sarcophilus*-size hypercarnivore (i.e., it was larger than *G. djadjinguli* and much larger than malleodectids) from the possibly late Miocene Wholly Dooley Site west of the Riversleigh World Heritage Area^20^. Its relationships to other dasyuromorphians, including other durophagous carnivores such as malleodectids, are unclear. Features that suggest it had a powerful bite include the short, wide, strongly-buttressed crown, hypertrophied protoconid, reduced metaconid and anteroposteriorly foreshortened talonid. Because lower molars for undoubted malleodectids have not been described, it is not yet possible to directly compare the features of the holotype of this new dasyuromorphian to malleodectids. Discovery of more complete lower and upper molars for the new hypercarnivore will be required to resolve the relationships of this taxon”.

These errors have now been fixed in the HTML and PDF versions of this Article.

