# Supplementary material for: A new family of bizarre durophagous carnivorous marsupials from Miocene deposits in the Riversleigh World Heritage Area, northwestern Queensland
Source: Sci Rep. 2016 May 27;6:26911. doi: 10.1038/srep26911 (PMC4882580; doi:10.1038/srep26911)
Supplement: Supplementary Information [file srep26911-s1.pdf]

Supplementary information for

A new family of bizarre durophagous carnivorous marsupials from Miocene deposits in the Riversleigh World Heritage Area, northwestern Queensland

M. Archer, S. J. Hand, K. H. Black, R. M. D. Beck, D. A. Arena, L. A. B. Wilson, S. Kealy, T.-t. Hung

1. Craniodental characters used in the phylogenetic analysis

Characters with asterisks represent plausible morphoclines and so were specified as ordered in the analysis.

1. Upper incisor number. Wroe & Musser (2001) ch.#1.
    0 5
    1 4

2. Morphology of C1. Wroe & Musser (2001) ch.#4.
    0 Caniniform
    1 premolariform

3. C1 alveolus. New.
    0 between premaxilla and maxilla
    1 entirely within maxilla

4. dP3 morphology. New.
    0 vestigial; no distinct cusps identifiable
    1 distinct paracone and metacone identifiable, but stylar shelf absent and protocone absent/weakly developed
    2 protocone present

5*. Height of P3. Wroe & Musser (2001) ch.#5.
    0 Higher crowned than P2
    1 subequal in height
    2 lower crowned than P2
    3 absent

6. Shape of P3 in occlusal view. Wroe & Musser (2001) ch.#6.
    0 Laterally compressed - much longer than wide
    1 bulbous and ovate - not markedly longer than wide

7*. Presence or absence of anterior and posterior crests on P3. New.
0 both anterior and posterior crests present and well-developed
1 only posterior crest well-developed
2 anterior and posterior crests absent or weakly-developed

8. Posterolingual cuspule present or absent on P3. Wroe & Musser (2001) ch.#7.
0 Absent
1 present

9*. Relative timing of eruption of P3. New.
0 after M4
1 simultaneous with M4
2 before M4 but after M3
3 simultaneous with M3
4 before M3

10. Relative size of paracone and metacone on M2-3. Modified from Wroe & Musser (2001) ch.#8. M1 was not used in scoring this character as it showed markedly different morphology from that of M2-3, which reduced consistency of character scoring.
0 Paracone and metacone equal or almost equal in size to metacone
1 metacone larger than paracone

11. Metacone on M4. Wroe & Musser (2001) ch.#9.
0 Present and distinct from metastylar corner of tooth
1 present but not distinct from metastylar corner of tooth
2 absent

12. Shape and orientation of the centrocrista. Modified from Wroe & Musser (2001) ch.#10 and ch.#11. These characters were combined because they are clearly non-independent, and also to result in character states that more closely correspond to the morphologies we observed. In doing so, character states taken from Wroe & Musser's (2001) character #10 are simplified, allowing less ambiguous scoring.
0 centrocrista straight, apex terminating close to level of talon basin
1 centrocrista weakly v-shaped
2 centrocrista strongly v-shaped
3 centrocrista incomplete, breaching the ectoloph
4 centrocrista straight, with apex well above talon basin

13. Preparacrista on M1. Modified from Wroe & Musser (2001) ch.#12. The original character was divided into two separate characters to reflect presence/absence and morphology of the preparacrista if present (see character 14).
0 present
1 absent

14*. Orientation of preparacrista on M1. Modified from Wroe & Musser (2001) ch.#12. Taxa lacking a preparacrista (character 13) were scored as inapplicable for this character.
0 M1 preparacrista oriented anterobuccally relative to long axis of the tooth
1 M1 preparacrista forms a near perpendicular angle with respect to the long

axis of the tooth

2 M1 preparacrista runs posterobuccally relative to long axis of the tooth

15. Relative lengths of M3 and M4 preparacristae. Wroe & Musser (2001) ch.#13.

0 M4 preparacristae shorter than or equal to that of M3
1 M4 preparacristae longer than that of M3

16*. Size of stylar cusp B on M3. Wroe & Musser (2001) ch.#17.

0 Large
1 small
2 absent

17*. Relative size of stylar cusp B and stylar cusp D on M2-3. Modified from Wroe & Musser (2001) ch.#18 and ch.#19. In an attempt to improve consistency of scoring, the two original characters were combined, and states were redefined to be less ambiguous.

0 stylar cusp D present and much larger than stylar cusp B
1 stylar cusp D present but smaller or subequal to stylar cusp B
2 stylar cusp D absent

18. Relationship of stylar cusp D to metacone. Wroe & Musser (2001) ch.#20.

0 Not appressed
1 appressed

19. 'Central cusp'. Wroe & Musser (2001) ch.#22.

0 absent
1 present

20. Lower incisor number. Wroe & Musser (2001) ch.#26.

0 4
1 3

21. i3 bilobed or not bilobed. Wroe & Musser (2001) ch.#27.

0 Not bilobed
1 bilobed

22. i2 morphology. Modified from Wroe & Musser (2001) ch.#29. The incisor examined for this character is the numerical second incisor, and is referred to here as i2, rather than i3 as it was in Wroe and Musser (2001).

0 Not staggered
1 staggered

23*. Height of p3 relative to p2. Wroe & Musser (2001) ch.#43.

0 p3 higher crowned than p2
1 subequal in height
2 smaller than p2
3 absent

24. Presence or absence of hypoconulid notch. Wroe & Musser (2001) ch.#30.

0 Present

1 absent

25. Entoconid size. Wroe & Musser (2001) ch.#41.
    0 Large
    1 reduced or absent

26. Size of metaconid on m1 relative to that of m2-4. Wroe & Musser (2001) ch.#32.
    0 Metaconid of m1 not reduced relative to that of m2-4
    1 Metaconid of m1 reduced relative to that of m2-4

27*. Size of metaconid in m2-4. Wroe & Musser (2001) ch.#33.
    0 Large
    1 reduced
    2 absent

28*. Size of paraconid in m1. Modified from Wroe & Musser (2001) ch.#34. The description of state 1 has been modified to improve scoring consistency.
    0 Large
    1 reduced, but still identifiable as a distinct cusp
    2 absent

29. Posterior cingulid in m1-3. Wroe & Musser (2001) ch.#36.
    0 present
    1 absent

30*. Anterior point of termination of the cristid obliqua in m3 with respect to carnassial notch formed by postprotocristid and metacristid. Wroe & Musser (2001) ch.#40.
    0 lingual to carnassial notch
    1 Beneath carnassial notch
    2 buccal to carnassial notch

31. Relative size of m4 to that of m3. Modified from Wroe & Musser (2001) ch.#42. The character states were changed to represent overall occlusal area rather than anteroposterior dimensions, in order to allow more consistent scoring.
    0 occlusal area of m4 smaller than that of m3
    1 occlusal area of m4 larger than or equal than that of m3

32*. Number of distinct cusps on m4 talonid. Wroe & Musser (2001) ch.#44.
    0 Three cusps
    1 two cusps
    2 one cusp

33. Presence or absence of orbital crest. Wroe & Musser (2001) ch.#45.
    0 Absent
    1 present

34. Maxillopalatine fenestrae. New (see Voss and Jansa, 2009).
    0 absent
    1 present

35. Palatine fenestrae. New (see Voss and Jansa, 2009).
0 absent
1 present

36. Anterior palatal fenestrae. (see Voss and Jansa, 2009).
0 absent
1 present

37. Presence or absence of complete posterolateral palatine foramen. Wroe & Musser (2001) ch.#48.
0 Present
1 absent

38. Presence or absence of accessory posterolateral palatine foramen. Wroe & Musser (2001) ch.#49.
0 Present
1 absent

39. Contribution of alisphenoid and periotic to primary foramen ovale. Wroe & Musser (2001) ch.#50.
0 Delimited by alisphenoid anteriorly and periotic part of the petrosal posteriorly
1 delimited by alisphenoid only

40*. Presence or absence of secondary foramen ovale formed by anteriorly directed strut of alisphenoid tympanic process. Wroe & Musser (2001) ch.#51.
0 Absent
1 present but incomplete
2 present and complete

41*. Presence or absence of secondary foramen ovale formed by mesial fold in alisphenoid tympanic process. Wroe & Musser (2001) ch.#52.
0 Absent
1 present but incomplete
2 present and complete

42*. Presence or absence of contribution to secondary foramen ovale by posteriorly directed strut in alisphenoid. Wroe & Musser (2001)
0 Absent
1 present but incomplete
2 present and complete

43. Ventral facial nerve canal. Modified from Wroe & Musser (2001) ch.#54. The original character states were simplified to simply presence and absence to ensure more consistent scoring. The identity of the bone(s) enclosing the canal has not been scored here.
0 Absent
1 present

44. Squamosal epitympanic sinus. Modified from Wroe & Musser (2001) ch.#55. The original character state descriptions used by Wroe & Musser (2001) mentioned size,

but here we have scored presence or absence only.
0 Absent
1 present

45. Bones contributing to hypotympanic sinus roof. Modified from Wroe & Musser (2001) ch.#56. The original character states 1, 2 and 3 were re-written slightly to reduce ambiguity.
0 alisphenoid and petrosal
1 alisphenoid only (i.e. separated from alisphenoid hypotympanic sinus by distinct petrosal ridge)
2 alisphenoid, petrosal and squamosal

46*. Size of alisphenoid tympanic process. Wroe & Musser (2001) ch.#57.
0 Absent
1 poorly developed (i.e. with alisphenoid hypotympanic sinus visible in external view)
2 well developed (i.e. extends posteriorly to reach posterior limit of alisphenoid contribution of alisphenoid hypotympanic sinus in ventral view)

47. Length of the internal jugular canal. Wroe & Musser (2001) ch.#60.
0 Does not extend anteriorly to the basisphenoid
1 extends to basisphenoid

48. Presence or absence of a well-developed posteroventral lip formed by a mesially directed process in the pars petrosa, enclosing the internal jugular ventrally. Wroe & Musser (2001) ch.#61.
0 Absent
1 present

49. Presence or absence of transverse canal. Wroe & Musser (2001) ch.#63.
0 Absent
1 present

50. Frontal-squamosal or alisphenoid-parietal contact on the lateral wall of the braincase. Modified from Wroe & Musser (2001) ch.#64 The character state descriptions were modified to reduce ambiguity.
0 alisphenoid-parietal contact.
1 Frontal-squamosal contact.

51*. Morphology of the rostral tympanic process of the petrosal. Modified from Wroe & Musser (2001) ch.#65. The original character (which referred to the rostral tympanic process of the petrosal as a 'tympanic wing of the periotic') was modified to reference the contact between the rostral tympanic process of the petrosal and alisphenoid as we found this too difficult to score consistently. Ordered.
0 Absent
1 present but small, not enclosing a sinus
2 present and large, enclosing a distinct sinus
.
52. Paroccipital process. Modified from Wroe & Musser (2001) ch.#66. Character states were simplified to presence and absence to ensure more consistent scoring.

0 Absent
1 Present

53. Morphology of foramen for the greater petrosal nerve. Modified from Wroe & Musser (2001) ch.#69. Wroe & Musser (2001) referred to this structure as the 'foramen lacerum medium', but it is less ambiguously referable to as the foramen for the greater petrosal nerve (see Wible, 2003: fig. 6).)
0 Distinct from primary foramen ovale
1 confluent with primary foramen ovale

54. Tubal foramen. Modified from Wroe & Musser (2001) ch.#70. The original character of Wroe & Musser (2001) has been subdivided into two separate characters here.
0 Absent
1 present

55. Morphology of tubal foramen. Modified from Wroe & Musser (2001) ch.#70. This character scores the shape of the tubal foramen. Taxa in which the tubal foramen is absent (character 54) were scored as inapplicable.
0 slit-like and incomplete anteroventrally
1 ovoid, with sulcus in pars petrosa
2 sulcus in alisphenoid tympanic process

56. Shape of nasals. From Wroe & Musser (2001) ch.#71.
0 Nasals posteriorly expanded
1 not posteriorly expanded

57. Maxilla-nasal contact. From Wroe & Musser (2001) ch.#72.
0 Maxilla-nasal contact longer than premaxilla-nasal contact
1 premaxilla contact longer than maxilla-nasal contact

58. Posterior extension of nasals. From Wroe & Musser (2001) ch.#73.
0 Nasals extend posteriorly beyond the anterior rim of the orbit
1 do not extend posteriorly beyond the orbit

59. Frontal-maxillary contact. From Wroe & Musser (2001) ch.#74.
0 Present
1 absent

60. Morphology of jugal. From Wroe & Musser (2001) ch.#75.
0 Jugal not Y-shaped
1 jugal Y-shaped

61. Antorbital fossa. Modified from Wroe & Musser (2001) ch.#76. This character now simply refers to presence or absence of the antorbital fossa.
0 absent
1 present

62. Presence or absence of prootic canal. Wroe & Musser (2001) ch.#77.
0 Present

1 absent

2. Molecular scaffold used in phylogenetic analysis in Newick format

(Pucadelphys_andinus,Andinodelphys_cochambensis,Mayulestes_ferox,((Caluromys_philander,(Didelphis_marsupialis,Marmosa_murina)),(Dromiciops_gliroides,((Echymipera_kalubu,Perameles_nasuta),(Myrmecobius_fasciatus,Thylacinus_cynocephalus,((Planigale_spp.,(Sminthopsis_murina,Sminthopsis_crassicaudata,Ningaui_spp.,Antechinomys_laniger)),((Phascogale_tapoatafa,Antechinus_flavipes,(Paramurexia_rothschildi,(Murexechinus_melanurus,(Murexia_longicaudata,Phascomurexia_naso)))),(Myoictis_melas,Parantechinus_apicalis,Pseudantechinus_bilarni,Pseudantechinus_macdonnellensis,Dasykaluta_rosamondae,(Dasycercus_cristicaudata,Dasyuroides_byrnei),((Neophascogale_lorentzii,Phascolosorex_dorsalis),(Sarcophilus_harrisii,(Dasyurus_hallucatus,(Dasyurus_maculatus,Dasyurus_albopunctatus))))))))))));

Topology is based on Jansa et al. (2014), Mitchell et al. (2014) and Westerman et al. (2015). These references are listed below in this supplementary information.

3. Data matrix in Nexus format

```
#NEXUS

Begin data;
      Dimensions ntax=50 nchar=62;
      Format datatype=standard symbols="01234" gap=-;
      Matrix
Pucadelphys_andinus             001?0010210101101000010000000020?0000010---
002000(01)00(01)10-0000000
Andinodelphys_cochambensis      00??00?0?10(01)01101000010000000200????010--
-002000100010-0000000
Mayulestes_ferox                000?0010211401101000001?010100110?000010---
002000001?10-0001000
Didelphis_marsupialis
00020010210101101000012000001210011000 1?110001001011?120000000
Caluromys_philander
0012200000010101100001200010120100000(01)000000010100111120000000
Marmosa_murina
00021010010201111000012000001201 01(01)0010000000101101 10??0000000
Dromiciops_gliroides
00120010(012)0200002200000000000012010100010000101200112011100000001
Djarthia_murgonensis
?0?20010?1020110101???2000000200?????????????????????????????(01)
Ankotarinja_tirarensis
?????????1?20{01}?0101???2000000200??????????????????????????????
Keeuna_woodburnei
?????????10201?1101???0000002?0???????????????????????????????
Yarala_burchfieldi
?0???01001120210000111(012)10000120201010100 2?0001?0101?1??000011?
Echymipera_kalubu
11100011(134)1030210100111(01)10000100101000 1?010000100111110-1110110
Perameles_nasuta
01(01)00011(012)10302101001111100001001110001102001120011 1110-1110110
Myrmecobius_fasciatus           101(01)20204----------1001-0010--
0010000100000112001021 10-000000?
```

```
Mutpuracinus_archibaldi
10??0010?1120111000????0100?021??100?1??000102011?21?11010000?
Barinya_wangala
10??0120?1120110000 1?12000000201110011010001020110211110000 00?
Sminthopsis_floravillensis
?????????1?20{01}11000????010000202??????????????????????????????
Sminthopsis_murina
1(01)??(01)0?0?1120111000111201000020201101100001112111121111110 0001
Sminthopsis_crassicaudata
11110010011201100001 11(12)000000102111011000011121111211111100001
Planigale_spp.
11?000101122001100011120101102020100110000111211102111100 00001
Ningaui_spp.
1(01)?10010(01)1220111000111201 0010{12}02011011000011121110 21?11110000?
Murexia_longicaudata
10110010?1120110000111100000020?01001101001112111021111000000?
Murexechinus_melanurus
1011(01)01001120111000111200001020001001100001112111021111 1000000
Paramurexia_rothschildi
101?0010011201110001111000010{12}0101001101011112111021111 0000000
Phascomurexia_naso
10101010011201120001 11(12)00001020101001101001112111021111 0100000
Myoictis_melas
1010201001120110000 1(01)12000020200(01)1(01)0010000?11211(01)021111000000?
Phascogale_tapoatafa
1011(012)010(01)1120111000101000000020011001100001112111021111 0000001
Antechinus_flavipes
1111101001120110000111200000002 02(01)1001100001112111021111 1100000
Parantechinus_apicalis
111?2010?122011{12}00010120110202011110???000?11211?021???0000001
Pseudantechinus_bilarni
10??0010?112(01)1100001013011010201011011000011121111211111 10000?
Pseudantechinus_macdonnellensis
111?(23)010?112(01)110000101(23)011010201(01)10011000011121110211111 10000?
Antechinomys_laniger
11?10010011201110001111010000202111011000011121111211111 100001
Dasycercus_cristicaudata        111?3---
012200110001(01)13011020202111011000011121110211110100001
Dasykaluta_rosamondae           101?3---
?1220011000111301101020211001100001112111021111 0000001
Dasyuroides_achilpatna          ?????????1221-
?1000????011020202????????????????????????????????
Dasyuroides_byrnei              1(01)103---11121-
1000010130110202011110 11(01)00011121110211111000001
Neophascogale_lorentzii
1010201021020210000111 2000000200(01)1(01)00100001112111?21111100000?
Phascolosorex_dorsalis
1010201001120?11000111200001020001(01)0110000111211102111100 00000
Dasyurus_hallucatus             10??3--0?1121-
11000101300101020 1?1?011000?11121110211110000001
Dasyurus_maculatus              10103--0?1221-
100101013001110202011 0110?10111211102111100 00001
Dasyurus_albopunctatus          10??3---
?1120011000101300102020101?0(01)102001112111021111000000?
Sarcophilus_harrisii            101?3--0?1221-
10010101311112(01)2021100110220111211102111100 00001
Muribacinus_gadiyuli
????0011?1110111000????00010020????????????????????????????????
Badjcinus_turnbulli             100??????1111-
110001?12001100101?1?0??00100002001?1100-000000?
Nimbacinus_dicksoni
```

```
10??0011?11101110001?10000100201110011?2100102001?1100-0?0000?
Thylacinus_macknessi
?????????1?400?{01}??0???0010200?01??????????????????????????????
Thylacinus_potens
????0010?11400?2{01}00????010??0?12?100??????????????????????????
Thylacinus_cynocephalus
1000001041140012000111001020 0?121100(01)11210010200111100-000000(01)
Ngamalacinus_timmulvaneyi
????0011?1?101?1000???000100201???????????????????????????????
Malleodectes_spp.
?0?10120?1?101?100???????????????1????????????????????????????
      ;
End;
begin paup;
set mstax = var;
outgroup Pucadelphys_andinus;
constraint scaffold (backbone) =
(Pucadelphys_andinus,Andinodelphys_cochambensis,Mayulestes_ferox,((Caluromys
_philander,(Didelphis_marsupialis,Marmosa_murina)),(Dromiciops_gliroides,((E
chymipera_kalubu,Perameles_nasuta),(Myrmecobius_fasciatus,Thylacinus_cynocep
halus,((Planigale_spp.,(Sminthopsis_murina,Sminthopsis_crassicaudata,Ningaui
_spp.,Antechinomys_laniger)),((Phascogale_tapoatafa,Antechinus_flavipes,(Par
amurexia_rothschildi,(Murexechinus_melanurus,(Murexia_longicaudata,Phascomur
exia_naso)))),(Myoictis_melas,Parantechinus_apicalis,Pseudantechinus_bilarni
,Pseudantechinus_macdonnellensis,Dasykaluta_rosamondae,(Dasycercus_cristicau
data,Dasyuroides_byrnei),((Neophascogale_lorentzii,Phascolosorex_dorsalis),(
Sarcophilus_harrisii,(Dasyurus_hallucatus,(Dasyurus_maculatus,Dasyurus_albop
unctatus))))))))))));
ctype ord: 5 7 9 14 16 17 23 27 28 30 32 40 41 42 46 51;
end.
```

4. References for above supplementary data

Jansa, S. A., Barker, F. K., & Voss, R. S. (2014). The early diversification history of didelphid marsupials: a window into South America's "splendid isolation". *Evolution, 68*(3), 684-695.

Mitchell, K. J., Pratt, R. C., Watson, L. N., Gibb, G. C., Llamas, B., Kasper, M., et al. (2014). Molecular phylogeny, biogeography, and habitat preference evolution of marsupials. *Molecular Biology and Evolution, 31*(9), 2322-2330.

Voss, R. S., & Jansa, S. A. (2009). Phylogenetic relationships and classification of didelphid marsupials, an extant radiation of New World metatherian mammals. *Bulletin of the American Museum of Natural History, 322*, 1-177.

Westerman, M., Krajewski, C., Kear, B. P., Meehan, L., Meredith, R. W., Emerling, C. A., et al. (2015). Phylogenetic relationships of dasyuromorphian marsupials revisited. *Zoological Journal of the Linnean Society, 176*(3), 686-701.

Wroe, S., & Musser, A. (2001). The skull of *Nimbacinus dicksoni* (Thylacinidae : Marsupialia). *Australian Journal of Zoology, 49*(5), 487-514.

5. Video clip of 3D model of maxilla QM F57925 segmented from micro-CT scan.
